# Microbial Considerations for the Permanent Geological Storage of CO_2_

**DOI:** 10.1111/1462-2920.70195

**Published:** 2025-10-23

**Authors:** Sophie L. Nixon, Leanne Walker, Rebecca L. Tyne

**Affiliations:** ^1^ Manchester Institute of Biotechnology, University of Manchester Manchester UK; ^2^ Department of Earth and Environmental Sciences University of Manchester Manchester UK

## Abstract

Carbon capture and storage (CCS) is a cornerstone strategy for achieving Net Zero emissions, yet the role of microbial life in subsurface CO_2_ storage remains underexplored. This mini‐review highlights the deep biosphere as a key but overlooked player in CCS operations across saline aquifers, depleted hydrocarbon reservoirs and basalt formations. It synthesizes evidence that microbial communities can both compromise and enhance CO_2_ storage via processes like methanogenesis, sulfidogenesis, corrosion and carbonate mineralization. Drawing on insights from hydrocarbon extraction and early CCS case studies, the review emphasizes the need for comprehensive microbial and geochemical monitoring to assess risks and harness potential benefits. The authors advocate for a holistic biogeochemistry toolkit and cross‐sector collaboration to ensure safe, effective and microbiologically informed CCS deployment.

## Graphical abstract

The impacts of microbiology on the storage of CO_2_ are poorly understood but could be critical to its safe geological disposal. We review evidence for microbial risks and opportunities, and argue for a holistic biogeochemical approach to monitoring, managing and potentially harnessing microbial activity in geological storage operations.

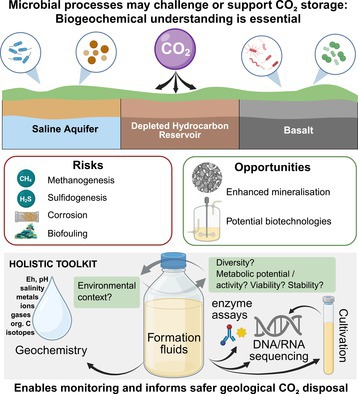

## 1 Introduction

**FIGURE 1:**
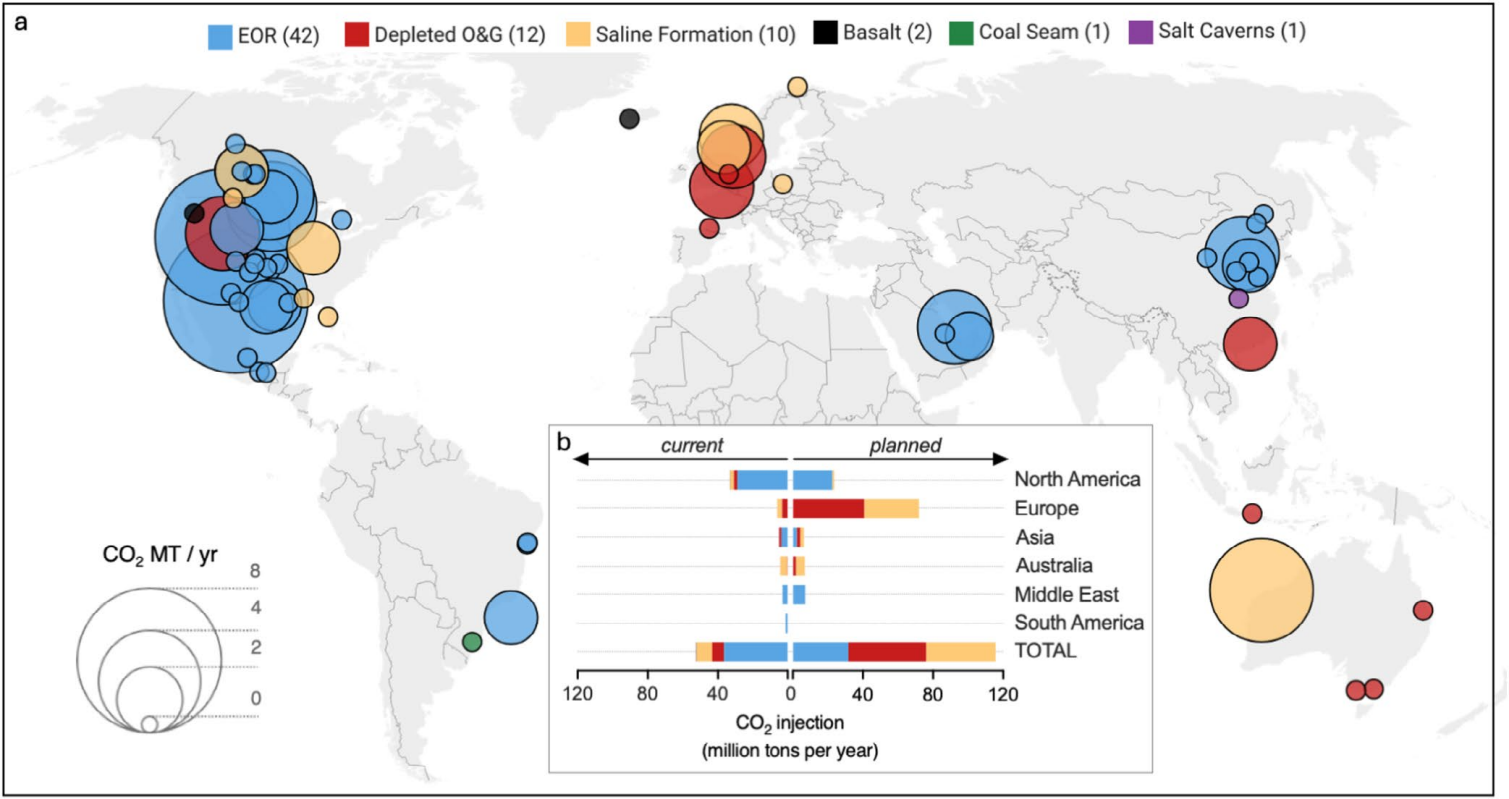
Current (a) and planned (b) global CCS operations by storage formation and CO_2_ injection volume (in millions of tons per year). EOR = enhanced oil recovery. Numbers in parentheses represent the number of CCS operations per formation type. Data were compiled from the SCCS database (https://www.sccs.org.uk, accessed September 2024), whereby current operations include ‘operational’, ‘pilot’ and ‘in build’ entries and planned operations include ‘planned’ and ‘in design’ entries (SCCS 2024).

Carbon capture and storage (CCS) is essential to achieve Net Zero by 2050 and avoid the worst impacts of climate change. In this process, CO_2_ is captured and injected into deep subsurface formations for permanent geological storage. The planning and application of CCS worldwide is increasing, especially within saline aquifers (Figure 1), which have the largest storage capacity. Depleted conventional oil and gas reservoirs are also increasingly being repurposed for CO_2_ storage. Yet critical knowledge gaps to achieve safe, permanent storage still exist, including the potential impact of the deep biosphere.

We are becoming increasingly reliant on the subsurface to mitigate the worst impacts of climate change (Amundson et al. 2024), and there has been significant research into the trapping mechanisms and geological conditions for safe geological CO_2_ storage (De Silva and Ranjith 2012; Matter et al. 2016; Espinoza and Santamarina 2017; Abba et al. 2019). However, despite the growing body of evidence which highlights the extent and nature of microbial life in the deep subsurface (Castelle and Banfield 2018; Ruff et al. 2024), and the significant body of research on the role of microbiology in hydrocarbon recovery (Gieg et al. 2011; Vigneron et al. 2017), much less attention has been paid to the role of microbiology in CCS operations. While the geomechanical and geochemical aspects of CCS have been the primary focus to date, the potential for microorganisms to directly or indirectly influence the fate of injected CO_2_—both positively and negatively—remains underexplored.

The advent of affordable DNA sequencing and downstream analytical approaches—in particular metagenomics—has created the unique opportunity to address the role of microbiology *prior to* widespread CCS implementation. We must be proactive in characterizing the potential risks to ensure safer and more sustainable CO_2_ sequestration. In so doing, we may also discover opportunities for safer CO_2_ storage and new utilization biotechnologies.

In this review, we highlight subsurface microbiology as an important consideration in the planning, implementation and monitoring of geological CO_2_ storage. This review focuses on prokaryotic microorganisms, which dominate the deep biosphere, though the role of microbial eukaryotes warrants further research. By applying a holistic biogeochemical toolkit, we argue that integrating microbial assessments into CCS design and monitoring efforts could improve the predictability, security and long‐term success of storage projects.

## 2 The Deep Biosphere and the Role of Subsurface Engineering

An estimated 70% of Earth's prokaryotic biomass resides in the subsurface (Whitman et al. 1998; McMahon and Parnell 2014). This microbiota is often viable and active (Pedersen 2000; D'Hondt et al. 2004; Schippers et al. 2005; Orsi et al. 2013; Lopez‐Fernandez et al. 2018; Bell et al. 2020) and is characterized by enormous phylogenetic diversity (Ruff et al. 2024). Indeed, much of the foliage on the modern tree of prokaryotic life derives from subsurface environments (Castelle and Banfield 2018). It is therefore unsurprising that human engineering activities in the deep subsurface impact—and are impacted by—the deep biosphere (Amundson et al. 2024).

Anthropogenic impacts on the deep biosphere related to hydrocarbon extraction are a good analogue for why it is essential to understand the role of the deep biosphere in CCS. For example, biogenic production of hydrogen sulfide, or ‘souring’, which is stimulated by seawater injection (Nemati et al. 2001), is a well‐documented and costly microbial problem for the oil industry and may also be a risk in CCS, especially where CO_2_ is stored in depleted oil reservoirs. Hydrogen sulfide promotes corrosion, degrades the value of the oil, and poses serious health risks to both oil industry workers and the environment alike, contributing billions of dollars in operational costs globally each year (Korenblum et al. 2010; Johnson et al. 2017). The scale of the problem has led to substantial scientific research into the associated microbiology (Gieg et al. 2011; Voordouw 2011; Bonifay et al. 2017). More recently, several studies have highlighted the potential for souring and other deleterious microbial activity within shale gas operations via the stimulation of fermentative bacteria that can metabolise injection fluid additives within high salinity shale formations (Cluff et al. 2014; Daly et al. 2016; Mouser et al. 2016; Booker Anne et al. 2017; Cliffe et al. 2020). While hydrocarbon extraction has clear impacts on the deep biosphere, the effects of other subsurface anthropogenic activities—including the permanent geological storage of CO_2_—on microbial communities remain largely unknown (Amundson et al. 2024).

## 3 Formations Targeted for CO_2_ Storage and Their Biogeochemical Characteristics

**FIGURE 2:**
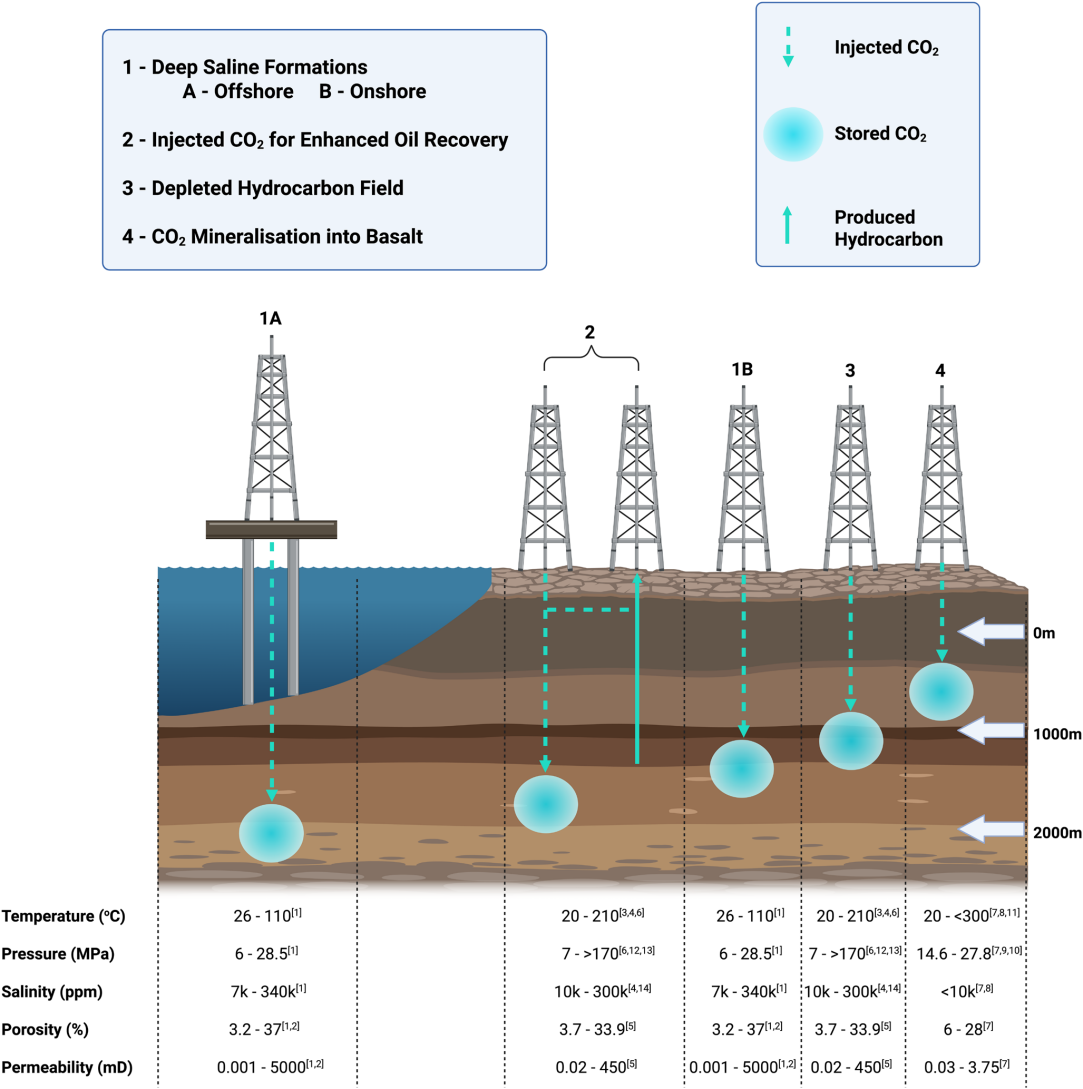
Key characteristics of the major formation types targeted for permanent geological CO_2_ storage. Created in BioRender. https://BioRender.com/i82t162 Adapted from: https://www.globalccsinstitute.com/wp‐content/uploads/2022/07/CCS‐Storage.jpg. Formation depths depicted in the figure above are representative of a range of typical reservoir depths for each type of CO_2_ storage formation. References cited: [1] (Michael et al. 2010), [2] (Mwakipunda et al. 2023), [3] (Nadeau et al. 2023), [4] (Dyer and Graham 2002), [5] (Chen et al. 2020), [6] (Wang et al. 2022), [7] (Snæbjörnsdóttir et al. 2014), [8] (Snæbjörnsdóttir et al. 2020). [9] (McGrail et al. 2010), [10] (McGrail et al. 2014), [11] (Clark et al. 2020), [12] (He et al. 2023), [13] (Ma et al. 2016), [14] (Sauerer et al. 2021).

The three main types of CO_2_ storage formations targeted in current and planned CCS operations are saline aquifers, depleted hydrocarbon reservoirs and basalt formations (Figures 1 and 2). These represent the most widely targeted formation types for large‐scale CCS, based on data compiled by Scottish CCS (https://www.sccs.org.uk, accessed September 2024) (SCCS 2024). Although we only discuss these formation types in detail below, other exploratory target formation types are gaining interest for potential CO_2_ storage and disposal, such as coal seams (Thielemann et al. 2004; Zhao and Wei 2022), salt caverns (Mwakipunda et al. 2024) and in mafic and ultramafic formations, such as peridotite (Matter et al. 2025).

Microbial activity in each formation type is influenced by geology, geochemistry, physiochemical conditions and diagenetic history. The native microbiota will differ between these formations; however, it is unknown how these inherent microbiome variations will be impacted during CCS operations. It is therefore essential that the baseline and ongoing diversity and metabolic activity of the microbial communities within these sites are profiled alongside geochemical changes to assess the potential risks and rewards of microbial activity throughout CO_2_ storage.

### 3.1 Saline Aquifers

A saline aquifer is defined as an onshore or offshore porous rock formation saturated with high salinity brine and bound by an impermeable caprock layer (Karvounis and Blunt 2021). Saline aquifers have the biggest CO_2_ storage potential (Figure 1) (De Silva and Ranjith 2012) and are not typically considered economically viable for other uses (Bentham and Kirby 2005; Luo et al. 2022).

The geochemical composition of fluids and physicochemical conditions between saline aquifers can vary considerably (Figure 2) (Michael et al. 2010; Mwakipunda et al. 2023). Salinity can range from 7113 ppm in the Haizume Formation in Japan to 340,000 ppm in the Alberta Basin, Canada (Michael et al. 2010), directly affecting microbial habitability in these environments. Temperatures similarly vary from as low as 26°C in the Alberta Basin, Canada, up to above 100°C in the deeper L. Tuscaloosa Formation, USA (Michael et al. 2010), and this can have a significant impact on the microbial communities that are able to colonise these subsurface environments (Michael et al. 2010; De Silva et al. 2015). However, the impacts of these geochemical factors on the subsurface microbiome are not well understood, as microbial community analysis before and after CO_2_ injection is not routine.

The potential for microbial activity in saline aquifers is likely to be driven primarily by salinity. We can look to hydraulically fractured shale gas wells as an example of salinity being the primary driver of microbial diversity and activity (Daly et al. 2016; Mouser et al. 2016). Numerous studies have identified remarkably similar microorganisms dominating geographically isolated and geologically distinct fractured shale formations (Cluff et al. 2014; Daly et al. 2016; Booker Anne et al. 2017; Cliffe et al. 2020; Hernandez‐Becerra et al. 2023). Although other factors, such as temperature, pressure and injection chemistry, play a role in the microbial communities that proliferate in these engineering subsurface environments (Cluff et al. 2014; Daly et al. 2016; Mouser et al. 2016), salinity appears to be the major factor affecting diversity and membership (Hernandez‐Becerra et al. 2023). For instance, the highest salinity produced fluids from fractured shales in the US harbor lower diversity specialist communities, dominated by halotolerant members of *Halanaerobium* and *Methanohalophilus* genera (Cluff et al. 2014, Daly et al. 2016, Mouser et al. 2016). In contrast, communities recovered from lower salinity produced waters from fractured shales in the UK and China are characterized by higher diversity communities, with less halotolerant lineages dominating, including *Marinobacter* spp., *Arcobacter* spp. and *Shewanella* spp. (Zhang et al. 2017; Cliffe et al. 2020; Hernandez‐Becerra et al. 2023). Clearly, the baseline salinity of any saline aquifer targeted for CO_2_ storage will act as a key driver for microbial diversity and membership. Lower salinity environments are expected to harbor higher phylogenetic and metabolic diversity, whereas higher salinity environments are expected to harbor lower diversity, specialized communities that may be limited in metabolic capabilities. More baseline characterization studies of target formations are needed to predict the impact of CO_2_ injection.

A small number of studies have attempted to assess the potential impact of CO_2_ storage in saline aquifer–inhabiting microorganisms. A pilot CO_2_ storage project in the Ketzin Aquifer, Germany, analysed baseline groundwater samples and demonstrated high microbial diversity and a community dominated by haloalkaliphilic fermentative bacteria prior to injection (Giese et al. 2009). Over a 5‐month CO_2_ injection period, a reduction in sulfate‐reducing bacteria, an increase in methanogenic archaea, and enhanced microbial activity were observed in the Ketzin Aquifer (Morozova et al. 2010). An increased abundance of *Comamonadaceae*, *Sphingomonadaceae* and methanogens has also been detected in samples from the Paaratte formation in the Otway basin after periods of supercritical CO_2_ injection, as well as a reduction in formation water pH, temperature and salinity (Mu et al. 2014; Mu and Moreau 2015). These examples suggest CO_2_ injection into saline aquifers results in a rapid shift towards chemolithotrophic and hydrogenotrophic microbial processes, as well as methane production (Tyne et al. 2023).

Notable examples of major offshore storage activities targeting saline aquifers for geological CO_2_ storage include the Sleipner Project, which has been injecting approximately 1 million tonnes of CO_2_ per year since 1996 into the Utsira formation beneath the North Sea, and the Northern Lights project, which intends to inject 1.5 million tonnes, scaling to 5 million tonnes, of CO_2_ per year into the Johansen Formation, also in the North Sea. Examples of onshore CO_2_ injection into saline aquifers include the Gorgon CO_2_ Injection Project on Barrow Island, Australia, which aims to inject up to 4 million tonnes of CO_2_ per year (though current sequestration rates are closer to 1.6 million tonnes); the Archer Daniels Midland project in Illinois, USA (> 1 million tonnes total CO_2_ storage to date), and the Santos and Beach Energy project, Australia, which has injected 1.7 million tonnes annually into the Cooper Basin. Significantly more CO_2_ storage projects are planned for saline aquifers worldwide (Figure 1), despite a critical knowledge gap on their native microbiota.

### 3.2 Depleted Oil and Gas Reservoirs

Depleted hydrocarbon assets already have impermeable caprocks for CO_2_ containment and can operate at high pressures common to CO_2_ injection operations (Ma et al. 2016; Wang et al. 2022; He et al. 2023). Additionally, these formations have existing infrastructure (including injection wells), significant reservoir modeling and historical operation data available to facilitate repurposing these sites for permanent geological CO_2_ storage (Dyer and Graham 2002; Chen et al. 2020; Nadeau et al. 2023).

The biogeochemical conditions of depleted hydrocarbon reservoirs are shaped by multiple factors, including reservoir location, formation geology, depth, geological age, amount of residual hydrocarbons present, injection and formation water salinity and previous operating conditions (Neff et al. 2011; Sauerer et al. 2021; Sharma et al. 2021). Microbial communities in these reservoirs predominantly consist of anaerobic microorganisms with diverse metabolic capabilities, such as sulfate reduction, methanogenesis, fermentation, acetogenesis, nitrate/nitrite reduction and iron cycling (Gieg et al. 2010; Meckenstock et al. 2016; Varjani and Gnansounou 2017). Residual hydrocarbons provide a readily available substrate for these subsurface microbial communities and drive processes such as sulfidogenesis, microbiologically influenced corrosion (MIC) and biofouling (Jones et al. 2008; Gieg et al. 2011; Skovhus et al. 2017; Scheffer et al. 2021). The presence of soluble hydrocarbons and volatile organic compounds can further stimulate microbial activity in depleted hydrocarbon reservoirs when repurposed for CO_2_ storage (Mayumi et al. 2013). Consequently, the emerging CCS industry should consider the potential risks of problematic microbial growth in these formations, particularly at sites with a history of microbial control issues during fossil fuel extraction.

Many oil and gas reservoirs have undergone CO_2_ injection for Enhanced Oil Recovery (EOR) (Luis et al. 2016; Zhao et al. 2016; Núñez‐López and Moskal 2019); Figure 1. However, only a few studies have addressed the impact of CO_2_ injection on subsurface microbial communities (West et al. 2011; O'Mullan et al. 2015). Injection of CO_2_ into a depleted oil reservoir in Northeast China was associated with an enrichment in genes for CO_2_ fixation, fermentative H_2_ production and methanogenesis (Liu et al. 2015). Additionally, acetoclastic methanogenesis has been observed in produced water samples incubated under high partial pressures of CO_2_ (Mayumi et al. 2013). These results suggest that CO_2_ injection could stimulate microbially mediated CO_2_ sequestration via carbonate formation or in biomass.

Key projects targeting depleted oil and gas reservoirs include the HyNet and Acorn CCS projects. HyNet will inject CO_2_ into three low‐temperature and pressure (31°C and 5–10 bar, respectively) depleted gas fields beneath the Irish Sea (Becker et al. 2021; Facchi et al. 2021). Acorn will inject into the depleted Goldeneye gas field in the North Sea (Spence et al. 2014; Lee et al. 2024). In both cases, storage will be greatly facilitated by the existing infrastructure and knowledge of these formations. The microbiological conditions in these formations, however, are not known.

### 3.3 Basalt

Although the least explored option to date (Figure 1), basalts have been targeted as compelling geological CO_2_ storage repositories (Matter et al. 2009; McGrail et al. 2010; Snæbjörnsdóttir et al. 2014). CO_2_ can be stored in basalts via liquid or supercritical CO_2_ injection, or dissolved in freshwater or seawater. If dissolved CO_2_ is injected into a basaltic formation, the CO_2_ will initially be contained via solubility trapping and a cap rock confining layer, followed by mineral trapping in the medium‐to‐long term (> 2 years) (Sigfusson et al. 2015; Matter et al. 2016; McGrail et al. 2017; Snæbjörnsdóttir et al. 2020). If supercritical CO_2_ is injected into a basalt formation, it first dissolves within the formation water and then follows the same trapping mechanisms as outlined above. Mineralization occurs at a much faster rate in basalts than in saline aquifer or depleted hydrocarbon formations due to the high concentration and availability of divalent cations which react with the CO_2_ to form carbonates (Xu et al. 2004; Raza et al. 2022). Two major CO_2_ storage and sequestration projects within basalt formations are the CarbFix project in Iceland and the pilot Wallula Basalt Project in Washington, USA (Matter et al. 2009; McGrail et al. 2014; McGrail et al. 2017; Clark et al. 2020) (Figure 1). The Wallula Basalt Project was a pilot demonstration and is no longer active; however, the Carbfix and CarbonQuest collaboration project in the USA will be in a similar location within North American basalt formations (Snæbjörnsdóttir et al. 2024).

Subsurface basalt formations are known to harbour diverse communities dominated by microorganisms that metabolise one‐carbon compounds, including methane. For example, water from the basalt formation at the Snake River Plain Aquifer, USA, contained both type I (*Methylomonas, Methylobacter* and *Methylocaldum*) and type II (*Methylocystis*) methanotrophic genera, indicating carbon cycling within the subsurface microbial community. Active methane cycling at various depths was also indicated by concentrations of over 1000 nM of methane which carried isotopic signatures of a biogenic origin (Newby et al. 2004). Fluids from Columbia River Basalt Group have also been shown to contain active microbial communities that can utilise hydrogen for methanogenesis and acetogenesis, and it has also been proposed that dissolved organic carbon could also be utilised by the biomass as an energy source (Stevens and McKinley 1995; Anderson et al. 1998). For example, groundwater sampling from the Wallula Basalt Pilot Project prior to CO_2_ injection determined that shallower depths had a higher diversity of archaeal genera, whereas samples collected from deeper wells contained a higher diversity of bacterial genera. These microbial communities were dominated by *Proteobacteria*, *Firmicutes* and *Actinobacteria*, as well as lineages implicated with hydrogen oxidation (*Hydrogenophaga*), methylotrophy (*Methylotenera*), methanotrophy (*Methylomonas*), dissimilatory iron reducection (*Geoalkalibacter*), sulfur oxidation (*Thiovirga*) and methanogenesis (*Methermicocccus*) (Lavalleur and Colwell 2013). Results from this study therefore suggest the potential for hydrogen and single carbon compounds to power microbial metabolism in this target formation. Taken together, these studies demonstrate clear potential for microbial communities in basalt formations to autotrophically produce and consume methane, and fix inorganic carbon, suggesting the injection of CO_2_ could stimulate these metabolic lifestyles in a geological storage application.

In contrast to these older basalt formations in the USA, CarbFix is actively injecting CO_2_ captured directly from air and dissolved in water into Icelandic basalts. These basalts are less than 0.8 million years old and associated with the Hengill volcanic system in the southwest of Iceland. Rich in divalent cations of calcium, magnesium and iron (ideal for CO_2_ mineralization into carbonates) and characterised by porosities of up to ~30%, these formations represent compelling storage formations for CO_2_ (Matter et al. 2016). Trias et al. (2017) explored the pre‐ and post‐microbial ecology of these formations and found the baseline community in the CarbFix target formation prior to CO_2_ injection to be diverse and active, primarily composed of microorganisms known to operate chemolithoautotrophic lifestyles that rely on inorganic carbon sources and generate energy through the cycling of sulfur, iron and hydrogen (Trias et al. 2017). Targeted enrichments for these populations confirmed viability, demonstrating that the CO_2_ storage formation hosts an active and diverse community. In response to CO_2_ injection, microbial communities became less diverse, attributed to both the direct introduction of CO_2_ as well as the associated pH drop, and a rise in the abundance of taxa implicated with CO_2_, sulfur and hydrogen utilisation was observed (Trias et al. 2017). These findings highlight the shift in community membership from metabolic generalists to specialists that CO_2_ injection induced and underscore the stimulating role that CO_2_ injection can have on basalt‐hosted communities.

Beyond basalt, ultramafic rocks such as peridotite, especially in serpentinizing environments, offer additional but less explored opportunities for CO_2_ mineralization and hydrogen generation (Boyd et al. 2024). These systems provide another point of intersection between microbiology, CO_2_ storage, and engineered H_2_ production.

## 4 Potential Risks and Opportunities From Microbial Activity in CO_2_ Storage

A wide range of microorganisms can use CO_2_ to drive metabolism. The direct microbial fixation of CO_2_ is known to occur via at least seven different pathways (including hydrogenotrophic methanogenesis), many of which are widespread in deep subsurface environments (Magnabosco et al. 2015; Simkus et al. 2016; Momper et al. 2017; Smith et al. 2019; Sahu et al. 2022). Carbon fixation is thought to be especially important to supporting subsurface microbial communities in the absence of organic compounds to fuel heterotrophy (Momper et al. 2017; Tyne et al. 2023). The near‐surface, naturally CO_2_‐rich Crystal Geyser in Utah further highlights the prominence of CO_2_‐fixing microorganisms in high CO_2_ subsurface environments (Emerson et al. 2016; Probst et al. 2017), where the Calvin–Benson–Bassham cycle and Wood–Ljungdahl pathway were found to be the dominant modes of fixation. When considered alongside evidence that CO_2_ injection can stimulate—rather than curtail—microbial communities (Morozova et al. 2010; Morozova et al. 2011; Ma et al. 2018), these studies suggest that CO_2_ injection and storage in the subsurface may support a wide array of carbon fixation pathways as a major route to primary productivity in target formations.

**FIGURE 3:**
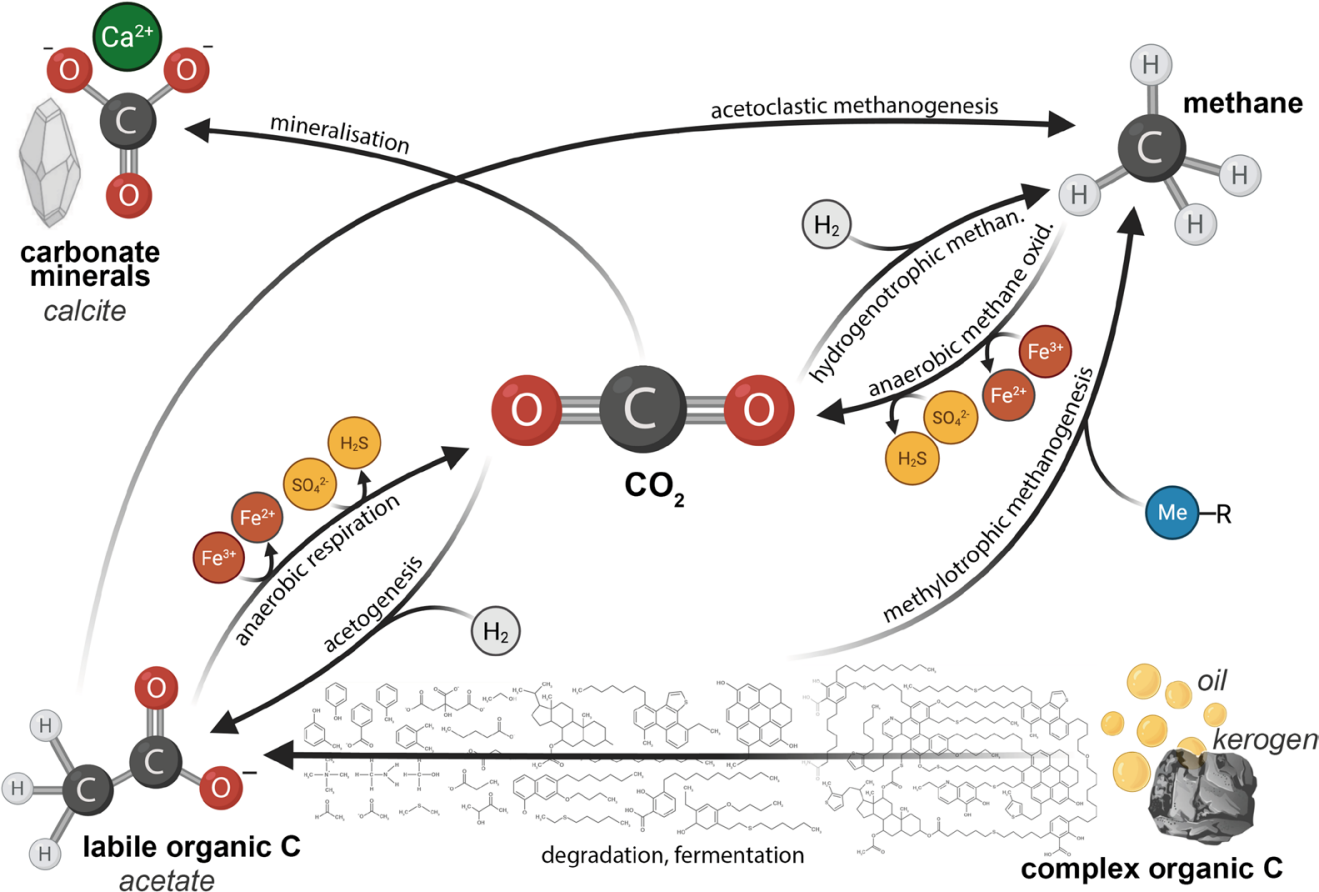
Potential metabolic reactions that deep biosphere communities could mediate in geological CO_2_ storage environments. Made using BioRender.

Although the primary metabolic reactions involving CO_2_ are well known, microbial CO_2_ utilisation (summarised in Figure 3) could feasibly result in a net gain or loss of CO_2_, production of methane and sulfide, and microbially influenced corrosion (MIC) (Tyne et al. 2021; Tyne et al. 2023). Such microbial activity could compromise the capacity, integrity and long‐term safety of CO_2_ storage sites. Alternatively, microbially enhanced CO_2_ sequestration could enhance the mineralization of CO_2_ within the subsurface, improve the overall success of CCS operations, and potentially uncover novel ways to convert waste CO_2_ into useful products (Matter et al. 2016; Trias et al. 2017; Bachleitner et al. 2023). The degree to which CO_2_ injection and storage drives these positive and negative impacts is explicitly linked to any assessment of risks to CO_2_ disposal into a given formation.

Perhaps the most pressing risk to address is from methanogenesis. Prior studies have implicated CO_2_ in subsurface hydrogenotrophic methanogenesis in a depleted hydrocarbon reservoir (Tyne et al. 2021, Tyne et al. 2023), raising the question of whether the CO_2_ we intend to store in the deep subsurface may in fact be partially converted to methane. Some may wonder whether it is irrelevant what form the injected CO_2_ takes in geological storage environments, so long as it remains in place over > 10,000‐year timescales. However, conversion of CO_2_ to methane poses several risks. Firstly, methane is more mobile than CO_2_ under subsurface conditions due to the reduced solubility of methane when compared to CO_2_ (Kastanidis et al. 2018; Sun et al. 2022). Given that methane is a significantly more potent greenhouse gas, any risk of leakage through compromised wellbores or faults and fractures will undo the climate mitigation effects of CCS. Secondly, methane is lower in density than CO_2_ and hence directly impacts the storage capacity of the formation. This would be compounded if geochemical conditions of the reservoir were altered to reduce CO_2_ trapping mechanisms. Other risks include reservoir pressurization due to increased gas volume and potential risk to workers from explosion.

Tyne et al. (2021) used isotopic fractionation and noble gas analyses to show that 13%–19% of CO_2_ injected into a depleted onshore oil reservoir in Louisiana, USA, was biologically converted to methane in less than 30 years (Tyne et al. 2021). This represents a rate of methanogenesis significantly faster than observed in natural analogues or experimental simulations and suggests full conversion to methane could occur over 100 of years. Further studies of CO_2_‐driven methanogenesis in the context of CCS operations are urgently needed to characterise whether conversion to methane is a carbon sink or whether it may in fact stimulate knock‐on methane oxidation (Figure 2), and under what conditions this process is favoured in different target formations (Tyne et al. 2021).

On the other hand, CCS may unlock opportunities to harness the microbial world to help achieve Net Zero emissions. Microbial activity in storage formations may enhance CO_2_ sequestration, either by catalyzing carbonate precipitation (Mitchell et al. 2013), enhancing solubility trapping (Emerson et al. 2016), conversion of CO_2_ and hydrogen into natural gas via bio‐methanation (Strobel et al. 2020), or by converting CO_2_ to biomass (Lavalleur and Colwell 2013). In addition, better characterizing the functional potential and activity of the diverse deep biosphere may bring to light new or more efficient modes of microbially mediated CO_2_ sequestration that can be leveraged via new biotechnologies. New tools such as single cell genomics (Lloyd et al. 2018) and isolation independent microbiome engineering (Rubin et al. 2022) have laid the groundwork for using microbial innovation in CO_2_ utilization to convert humankind's waste products into useful commodities without the need for fossil fuel feedstocks.

Many of the environmental parameters that influence microbial metabolism—including pH, temperature and redox state—also directly affect carbonate precipitation and stability, creating coupled biogeochemical feedback loops in CO_2_ storage sites. The redox state of the target formation could be influenced by operational parameters such as CO_2_:rock ratio or co‐injection of oxygenated water. Coupled with tools such as reaction path thermodynamic modelling, these strategies could help predict and mitigate undesirable microbial processes while priming the system for optimal CO_2_ mineralization.

## 5 The Need for a Holistic Biogeochemistry Toolkit

The potential for the various metabolic processes mapped out in Figure 3 will depend on the characteristics of the target formation, spanning physical (temperature, pressure, porosity), chemical (salinity, pH, organic matter and H_2_ content, concentration of sulfur and iron species), and biological (native and introduced microbiota) factors. The impact of some such factors is relatively easy to predict. For instance, formations that are at depths where temperatures far exceed the upper limit for life (122°C; Takai et al. 2008) are unlikely to pose a microbiological risk to CCS. However, most formation types are characterised by habitable characteristics (Figure 2). The combination of these attributes will determine whether certain pathways are likely to occur, and hence, without holistic datasets for target formations, it is impossible to develop a generalisable predictive framework to effectively evaluate risk.

Some CCS operations (such as the Gorgon project in Australia) generate significant volumes of produced fluids. Such fluids carry with them the geochemical and microbiological fingerprint of the reservoir their derive from (Cluff et al. 2014), and can even harbour microorganisms that remain viable long after production (Cliffe et al. 2020). CCS produced fluids will not only hold clues of in situ activity that can be studied using geochemical and microbiological approaches, but they may also contain harmful microbial by‐products or biogenic greenhouse gases, for example methane and hydrogen sulfide. It is thus crucial that both downhole and produced fluids from target CO_2_ storage sites are geochemically and microbiologically screened prior to, and throughout, CO_2_ injection and storage to determine the native microbiota inhabiting these reservoirs, and to predict how these microbial communities may respond to CO_2_. This screening ensures that chemical treatments and metallurgy specifications are evaluated as part of a comprehensive microbial risk framework, mitigating potential impacts on system integrity and performance.

To fully understand and hence predict microbiological impacts in CCS requires the application of a holistic, systems‐level toolkit to characterise the biogeochemistry of target formations. Such a toolkit must be capable of assessing the number, viability and metabolic capabilities of the microbial communities inhabiting storage reservoirs, and the physico‐chemical conditions under which the impacts of their activity on permanent geological storage may become positive or negative. Without this comprehensive microbial surveillance, any assessment of risk remains incomplete.

## 6 Concluding Remarks

CCS will continue to ramp up at a time when microbiologists have unprecedented access to molecular tools that can adequately assess microbial risks. Yet, achieving sufficient microbial monitoring is not only a case of access to tools; it relies on multisector collaboration, bringing together scientific researchers who can deploy and interpret results from a holistic biogeochemical toolkit, CCS operators who can make available access to relevant subsurface samples, and legislators who must incorporate any microbial risks into operational requirements. Such collaborations can be challenging to establish and maintain, and are currently lacking, but are nonetheless essential. We hope this minireview serves to encourage such collaborations to emerge in order that CCS is deployed as a safe and urgently needed climate mitigation strategy in decades to come. In so doing, the hidden traits of the unseen majority of prokaryotic life will be unearthed, paving the way for new biotechnologies, a greater understanding of the limits of life, and the potential habitability of environments beyond Earth.

## Author Contributions

S.L.N., L.W. and R.L.T. conceived the idea for the work. L.W. made figure 2, S.L.N. made figures 1 and 3. S.L.N. drafted the manuscript with contributions from L.W. and R.L.T.

## Conflicts of Interest

The authors declare no conflicts of interest.

## Data Availability

The data that support the findings of this study are available in Scottish Carbon Capture & Storage repository at http://sccs.org.uk/resources/global‐ccs‐map. These data were derived from the following resources available in the public domain: http://sccs.org.uk/resources/global‐ccs‐map, http://sccs.org.uk/resources/global‐ccs‐map.
